# Effect of Discontinuation of Renin Angiotensin-System Inhibitors in Patients With Advanced Chronic Kidney Disease: A Meta-Analysis

**DOI:** 10.7759/cureus.37813

**Published:** 2023-04-19

**Authors:** Husnain Naveed, Gayathri Tirumandyam, Gautham Varun Krishna Mohan, Sawara Gul, Shahid Ali, Alveena Siddiqui, Zoilo K Suarez, Areeba Khan

**Affiliations:** 1 Medicine and Surgery, Shifa Tameer-E-Millat University Shifa College of Medicine, Islamabad, PAK; 2 Internal Medicine, Siddhartha Medical College Vijayawada, Tirupathi, IND; 3 Internal Medicine, Tirunelveli Medical college, Tirunelveli, IND; 4 Internal Medicine, Lady Reading Hospital, Peshawar, PAK; 5 Internal Medicine, Khyber Medical College, Peshawar, PAK; 6 Pediatrics, Jinnah Sindh Medical University, Karachi, PAK; 7 Internal Medicine, Florida Atlantic University Charles E. Schmidt College of Medicine, Boca Raton, USA; 8 Critical Care Medicine, United Medical and Dental College, Karachi, PAK

**Keywords:** meta-analysis, continuation, discontinuation, chronic kidney disease, ras inhibitors

## Abstract

Renin-angiotensin system inhibitors (RAS) inhibitors include angiotensin receptor blockers (ARBs) and angiotensin-converting enzyme (ACE) inhibitors decrease proteinuria, progression of chronic kidney disease (CKD), and protect against heart failure hospitalizations and cardiovascular events. There is uncertainty about the appropriate time for discontinuing ARB and ACE inhibitor treatment in patients with low estimated glomerular filtration rate (eGFR). In the present meta-analysis, we examined the effect of RAS inhibitor discontinuation on clinical outcomes in patients with advanced CKD compared to the continuation of RAS inhibitors. Two authors conducted electronic database searches in PubMed, the Cochrane Library, and Excerpta Medica Database (EMBASE) for relevant studies published from the inception of the databases to March 15th, 2023, using the following combination of keywords or key terms: "Renin-angiotensin-system," "angiotensin-converting-enzyme inhibitors", "Angiotensin receptor blockers," and "advanced chronic kidney disease." Primary outcomes assessed in this meta-analysis included cardiovascular events. Secondary outcomes assessed included all-cause mortality and end-stage kidney disease (ESKD). A total of four studies were included in this meta-analysis. The pooled analysis showed that cardiovascular events were significantly higher in patients in the discontinuation group compared to the continuation group (HR: 1.38, 95% CI: 1.21-1.58), ESKD was also significantly higher in the discontinuation group (HR: 1.29, 95% CI: 1.18-1.41). No significant differences were reported between the two groups in all-cause mortality. In conclusion, our meta-analysis provides evidence that continuation of RAS inhibitors could be beneficial in patients with advanced CKD, as it is associated with less risk of cardiovascular events and ESKD.

## Introduction and background

Chronic kidney disease (CKD) affects 8% to 16% of people around the world and it is often underrecognized by clinicians and patients [1-2], defined by a glomerular filtration rate (GFR) of less than 60 mL/min/1.73 m2, albuminuria of at least 30 mg per 24 hours or renal damage markers like structural abnormalities or hematuria continuing for more than three months [3]. While diabetes and/or hypertension are the leading causes of CKD worldwide, in Asia, sub-Saharan Africa, and many developing countries, other factors such as glomerulonephritis, infection, and exposure to environmental factors like air pollution, herbal remedies, and pesticides are also frequent contributors to CKD [4].

In patients with mild or moderate chronic kidney disease (CKD), the use of renin-angiotensin-aldosterone system (RAAS) inhibitors can decrease blood pressure, slow decline in the estimated glomerular filtration rate (eGFR), and decrease proteinuria [5]. Renin-angiotensin-aldosterone system (RAAS) inhibitors are a group of drugs that act by inhibiting the renin-angiotensin-aldosterone system (RAAS) and include angiotensin-converting enzyme inhibitors (ACE inhibitors), angiotensin-receptor blockers (ARBs), and direct renin inhibitors. They are the mainstays of treatment for heart failure with reduced ejection fraction (HFrEF), chronic kidney disease (CKD), hypertension, and coronary artery disease [6-8]. However, the benefits of these agents must be weighed against the possible risks, such as hyperkalemia, acute kidney injury, and a reduction in the estimated glomerular filtration rate (eGFR) [9]. The risks of the adverse events mentioned above are especially relevant in patients with lower eGFR.

It is believed that renin-angiotensin system (RAS) inhibitors slow down the progression of CKD by reducing proteinuria. However, there is an ongoing debate about the benefits of using RAS inhibitors in advanced stages of CKD (stage 4 or 5). A meta-analysis conducted on a general population showed that the advantage of lower urinary albumin excretion for reducing the risk of cardiovascular mortality disappeared in patients with an eGFR of less than 30 mL/min per 1.73 m2 [10].

Additionally, using RAS inhibitors in individuals with significantly impaired kidney function may have adverse effects, such as an increased risk of hyperkalemia, hemodynamic effects that can worsen kidney function, and a weakened response to acute kidney injury events [11]. Therefore, medical guidelines suggest reducing the dosage or discontinuing ACE inhibitors or ARBs in individuals with stage 5 CKD (eGFR <15 mL/min per 1.73 m2) if there is symptomatic hypotension or uncontrolled hyperkalemia [12]. However, the benefits and drawbacks of initiating or discontinuing RAS inhibitors in patients with advanced CKD are not clear. Furthermore, not many studies have been conducted that compared the continuation and discontinuation of RAS inhibitors in patients with CKD.

This meta-analysis aims to determine the effect of RAS inhibitor discontinuation on clinical outcomes in patients with advanced CKD compared to the continuation of RAS inhibitors. It conducted a pooled analysis of available studies to assess the association of RAS inhibitor discontinuation with the risk of major cardiovascular events, all-cause mortality, and end-stage kidney disease among individuals receiving RAS inhibitor therapy whose eGFR reduced to below 30 mL/min/1.73 m2.

## Review

Methodology

This meta-analysis was conducted in accordance with the Preferred Reporting Items for Systematic Reviews and Meta-Analyses (PRISMA) statement.

Search Strategy

Two authors (GT and SG) conducted electronic database searches in PubMed, the Cochrane Library, and Excerpta Medica Database (EMBASE) for relevant studies published from the inception of the databases to March 15th, 2023, using the following combination of keywords or key terms: "Renin-angiotensin-system," "ACE inhibitors," "Angiotensin receptor blockers," and "advanced chronic kidney disease." These key terms were combined using Boolean algebra operators (and, or). Medical subject headings (MeSH) terms were also used to increase the sensitivity of the search for finding more relevant studies. The reference lists of included articles were also manually screened to identify potentially eligible studies.

All potentially eligible studies were imported to ENDNOTE version X9. After removing duplicates, two authors independently screened the studies using titles and abstracts followed by a full-text detailed assessment of eligible studies. Any disagreements in the process of searching and study selection were resolved via discussion or involvement of the principal investigator if required.

Eligibility Criteria

The inclusion criteria were as follows: (a) participants aged 18 years or older; (b) advanced CKD (chronic kidney disease in which there is a severe reduction in glomerular filtration rate (GFR < 30 ml/min)); (c) RAS inhibitors, including either angiotensin-converting enzyme inhibitors (ACEI) or ARB administration; (d) at least one outcome was reported. We excluded studies that compared RAS inhibitors with any other antihypertensive drugs, non-original studies included case reports, editorials, and reviews, and were conducted in patients under the age of 18 years. We included only those studies published in the English language.

Data Extraction and Quality Assessment of the Included Studies

The characteristics of the included studies and outcomes of interest were extracted and managed using an Excel Spreadsheet. The characteristics of the studies included first author name, year of publication, study design, study groups, sample size, and follow-up duration. Primary outcomes assessed in this meta-analysis included cardiovascular events (composite of stroke, myocardial infarction, cardiovascular mortality, and heart failure hospitalization). Secondary outcomes assessed included all-cause mortality and end-stage kidney disease (ESKD). To assess the quality of studies, Newcastle-Ottawa Scale (NOS) was used for cohort studies, and the Cochrane Collaboration tool was adopted for randomized control trials (RCT). 

Statistical Analysis

We used a hazard ratio (HR) with a 95% confidence interval (CI) to determine the effect size of dichotomous outcomes in this meta-analysis. The pooled HR was calculated with the random-effect model if the heterogeneity was more than 50%; otherwise, the fixed-effect model was used. Heterogeneity among the study results was assessed using I-square statistics, and the value of I-square was divided into three levels, including low heterogeneity (<25%), moderate heterogeneity (25-75%), and high heterogeneity (>75%). We performed subgroup analysis based on the study design (observational studies and RCTs). Sensitivity analysis was also performed by removing a study with a high cardiovascular events rate and all-cause mortality. Review Manager 5.4.0 (Cochrane Collaboration, Oxford, UK) was used for statistical analysis. 

Results

**Figure 1:**
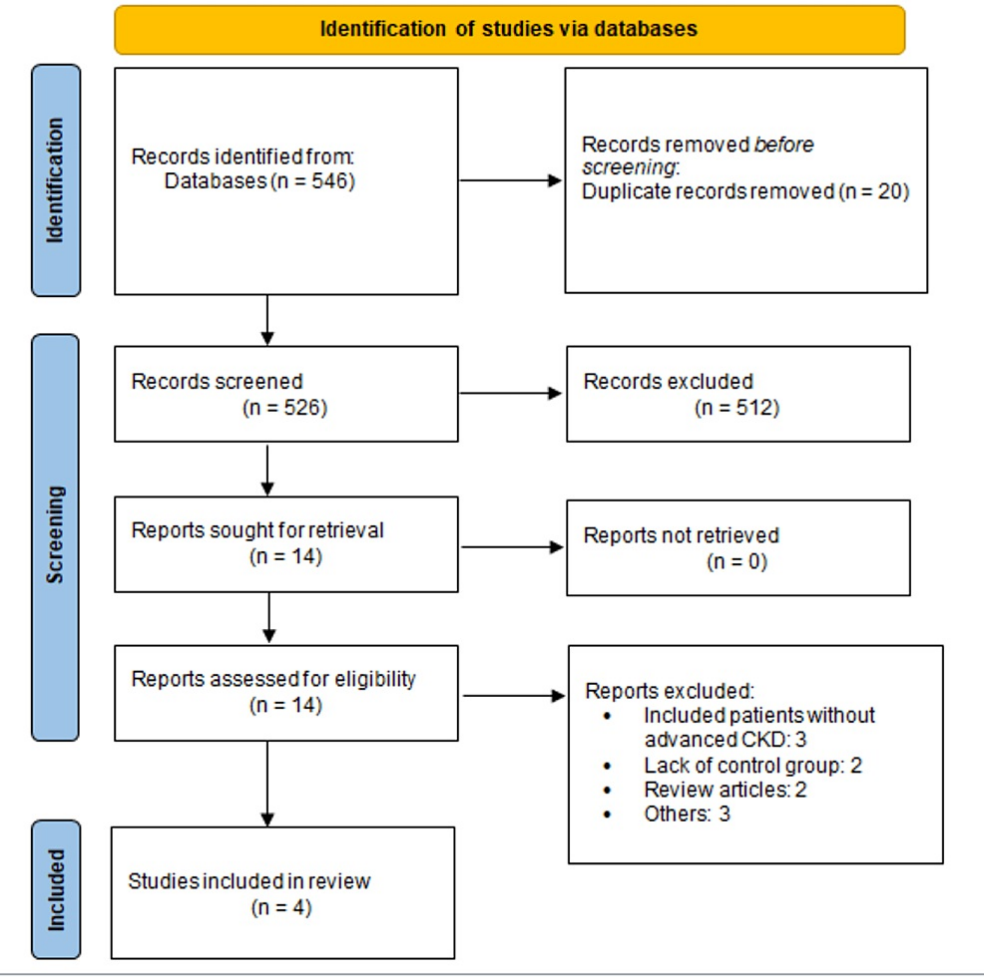
PRISMA flowchart of selection of studies

As shown in Figure 1, 546 articles were identified from the electronic search. Thereafter, 512 articles were excluded based on abstracts and title screening. Full texts of 14 articles were obtained and detailed assessment of inclusion and exclusion criteria were performed. Based on full-text, four studies were included in this meta-analysis. Table 1 shows the characteristics of included studies. Out of four studies, three were observational and one was RCT. Table 2 shows a quality assessment of included studies.

**Table 1:** Characteristics of included studies NR: Not reported; RCT: Randomized-control trial

Author Name	Year	Study Design	Groups	Sample Size	Follow-up Time	Mean Age (Years)	Male (%)	Diabetes (%)
Bhandari et al [13]	2022	RCT	Continued	205	36 Months	NR	69 vs 68	38 vs 36
			Discontinued	206				
Fu et al [14]	2021	Retrospective cohort	Continued	7971	60 Months	NR	NR	NR
			Discontinued	7078				
Qiao et al [15]	2020	Retrospective cohort	Continued	1205	60 Months	73.3 vs 73.1	41.4 vs 42.2	48.9 vs 47.5
			Discontinued	1205				
Yang et al [16]	2022	Prospective cohort	Continued	8634	6 Months	73.3 vs 73.3	48.2 vs 48.2	100 vs 100
			Discontinued	1766				

**Table 2:** Quality assessment of included studies

Quality Assessment for Retrospective Studies						
Study ID	Selection	Comparibility	Outcome	Overall		
Fu et al [14]	3	2	2	Good		
Qiao et al [15]	2	1	2	Fair		
Yang et al [16]	3	2	2	Good		
Quality Assessment for Randomized control trial						
Study ID	Selection Bias	Performance	Attrition	Reporting	Other	Overall
Bhandari et al [13]	No	Yes	No	No	Unclear	Moderate

Comparison of RAS Inhibitors Between Continuation and Discontinuation of Cardiovascular Events and All-cause Mortality

**Figure 2:**
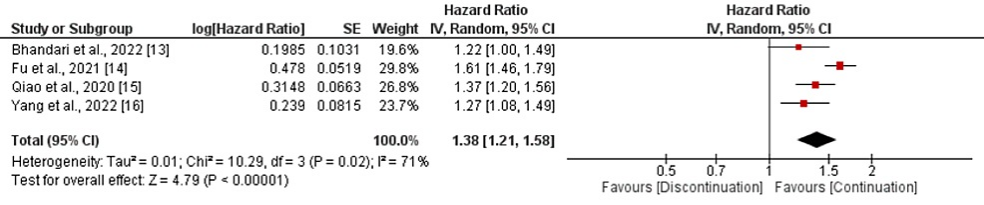
Comparison of risk of developing cardiovascular events between continuation and discontinuation groups Sources: References  [13-16]

Four studies reported cardiovascular events and the pooled cardiovascular was 29.90% and 47.66% in the continuation group and the discontinuation group respectively, and the hazard of developing cardiovascular events was significantly higher in patients in the discontinuation group compared to the continuation group (HR: 1.38, 95% CI: 1.21-1.58) with moderate heterogeneity among the study results (I-square: 71%, p-value: 0.02) as shown in Figure 2.

**Figure 3:**
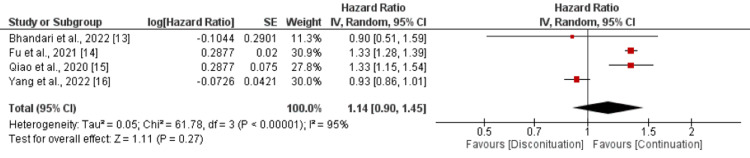
Comparison of risk of developing all-cause mortality between continuation and discontinuation groups Sources: References [13-16]

Four studies reported all-cause mortality and according to the pooled results hazard of all-cause mortality was higher in patients in the discontinuation group compared to the continuation group but the difference is statistically insignificant (HR: 1.14, 95% CI: 0.90-1.45) with high heterogeneity among the study results (I-square: 95%, p-value < 0.00001) as shown in Figure 3.

Comparison of RAS Inhibitors Between Continuation and Discontinuation of ESKD

**Figure 4:**
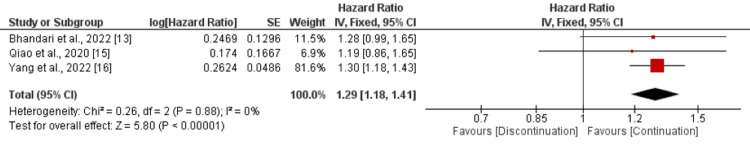
Comparison of risk of developing ESKD between continuation and discontinuation groups Sources: References [13, 15-16]

As shown in Figure 4, the pooled results show that the hazard of ESKD was significantly higher in the discontinuation group compared to the continuation group (HR: 1.29, 95% CI: 1.18-1.41) with no heterogeneity (I-square: 0%, p-value: 0.88) as shown in Figure 4.

Subgroup Analysis

Subgroup analysis based on the study design (observational and RCT) showed both RCT and observational studies supported that the hazard of cardiovascular events was significantly higher in the discontinuation group compared to the continuation group. Additionally, pooled analysis of observational studies showed the risk of mortality was lower in the continuation group. However, the difference was statistically insignificant. Moreover, subgroup analysis showed a high hazard of ESKD in patients in the discontinuation group in both RCT and observational studies but in RCT the difference was statistically insignificant as shown in Table 3.

**Table 3:** Subgroup Analysis RCT: Randomized-control trials; HR: hazard ratio; CI: confidence interval; ESKD: End-stage kidney disease; HR: hazard ratio; CI: confidence interval; NA: Not applicable * Significant at p-value<0.05

Outcome	Groups	HR (95% CI)	I-square
Cardiovascular events	Observational	1.46 (1.36-1.57)*	73%
	RCT	1.22 (1.00-1.49)	NA
All-cause mortality	Observational	1.18 (0.91-1.52)	97%
	RCT	0.90 (0.51-1.59)	NA
ESKD	Observational	1.29 (1.18-1.41)*	0%
	RCT	1.28 (0.99-1.65)	NA

Sensitivity analysis showed that removing the study conducted by Fu et al. [14] (higher mortality and cardiovascular outcome rates) in both the continuation and discontinuation group reduced heterogeneity, and the reduction in hazards of cardiovascular events but it remained statistically significant (HR: 1.31, 95% CI: 1.19-1.43, I-square: 0%). The reduction of mortality was also reported and it remained statistically insignificant (1.07, 95% CI: 0.79-1.45, I-square: 6%).

Discussion

To the best of our knowledge, this is the first meta-analysis comparing the effects of continuing and discontinuing RAS inhibitors in patients with CKD. We found that continuing RAS inhibitors could be beneficial in patients with advanced CKD, as it is associated with a lower risk of cardiovascular events and ESKD. The benefits of continuing RAS inhibitors were shown in both observational studies and RCTs.

A meta-analysis conducted by Xie et al. showed that the use of ARB or ACEI therapy was associated with a decreased risk of cardiovascular events and renal failure in patients with CKD [17]. Similarly, an observational cohort study conducted in the United States in individuals with CKD found survival benefits of RAS inhibitors across all levels of eGFR, including eGFR < 30 mL/min/1.73 m2 [18]. A study was conducted on 224 patients with serum creatinine levels ranging from 3.1 to 5.0 mg/dL who were randomly assigned to receive either benazepril or a placebo. The study found that benazepril resulted in a 43% lower risk of a combination of events such as doubling of serum creatinine level, end-stage kidney disease (ESKD), or death. Additional analyses of two other trials, the Reduction of Endpoints in NIDDM with the Angiotensin II Antagonist Losartan (RENAAL) and the Ramipril Efficacy in Nephropathy (REIN), indicated that ACE-I or ARB therapy may be beneficial for individuals with low GFRs [19-20].

A study conducted by Nakayama et al. examined the impact of continuing versus stopping or not using RAS inhibitors on the incidence of unplanned dialysis in patients with advanced CKD [20]. In a retrospective analysis, the study found that continuing RAS inhibitor therapy was associated with a lower incidence of unplanned dialysis initiation [21]. The authors suggest that with the development of potassium-binding therapy, RAS inhibitors are now better tolerated in advanced CKD patients. They propose that until randomized trials confirm any negative effects of RAS inhibitors on renal outcomes, it is reasonable to use these agents in patients with advanced CKD [22]. An RCT conducted to determine the impact of discontinuation of RAS inhibitors found that it is not associated with a significant improvement in the long-term rate of decrease in the eGFR. The study also reported an increased number of cardiovascular events and ESKD in the discontinuation group compared to the continuation group in patients with advanced CKD [13].

Hyperkalemia is one of the adverse events of RAS inhibitors in patients with advanced CKD. The study conducted by Yang et al. found that the overall incidence of the first hyperkalemia event was 99.8 per 1000 patient-years [16]. The rates were 101 and 99.6 per 1000 patient-years in the discontinuation and continuation groups respectively. It can be concluded that the risk of hyperkalemia is insignificant between the two study groups. However, we need more data from prospective clinical trials assessing the risks of withdrawing RAS blocker treatment in patients with advanced CKD.

Considering the uncertainty and the lack of clear guidelines, the prudent approach can be an evaluation of the risk-to-benefit ratio of maintaining RAS blockade on an individual basis. The discontinuation of RAS inhibitors should only be considered in specific cases of serious clinical issues, such as a significant decrease in eGFR severe hypokalemia that cannot be easily corrected, symptomatic hypotension, severe acidosis in the context of very low GFR, and possibly in the elderly population.

It is recommended that future research should focus on conducting more randomized controlled trials (RCTs) to provide stronger evidence on the efficacy of the continuation of RAS inhibitors in patients with advanced CKD. While the present meta-analysis showed that continuing RAS inhibitors may be beneficial in reducing the risk of cardiovascular events and ESKD, most of the evidence was extracted from observational studies. Further RCTs are needed to confirm these findings and provide more robust evidence.

Study Limitations

The present meta-analysis has certain limitations. Firstly, findings were predominantly extracted from observational studies. More RCTS are required to strengthen the evidence about the efficacy of the continuation of RAS inhibitors in advanced CKD patients. Secondly, ARBs and ACEI inhibitors cause different feedback in the RAAS; therefore they could exert different effects on inflammation and even on the clinical outcome. In the present meta-analysis, we were not able to perform subgroup analysis based on different RAS drugs due to a lack of data in the included studies.

## Conclusions

In conclusion, our meta-analysis provides evidence that continuation of RAS inhibitors could be beneficial in patients with advanced CKD, as it is associated with less risk of cardiovascular events and ESKD. However, the difference in all-cause mortality between the continuation and discontinuation groups was statistically insignificant. Our findings suggest that RAS inhibitors should be continued in patients with advanced CKD, and discontinuation of these agents should be carefully considered, as it may increase the risk of cardiovascular events and ESKD. Further large-scale randomized controlled trials are needed to confirm our findings and provide more definitive evidence.
